# LL-37 stimulates the functions of adipose-derived stromal/stem cells via early growth response 1 and the MAPK pathway

**DOI:** 10.1186/s13287-016-0313-4

**Published:** 2016-04-19

**Authors:** Yoolhee Yang, Hyunju Choi, Mira Seon, Daeho Cho, Sa Ik Bang

**Affiliations:** Department of Plastic Surgery, Samsung Medical Center, Sungkyunkwan University School of Medicine, Seoul, Korea; Department of Life Science, Sookmyung Women’s University, Seoul, Korea; Bio-Med Translational Research Center, Samsung Medical Center, Seoul, Korea

**Keywords:** LL-37, Mesenchymal stem cells, Adipose-derived stromal/stem cells, Early growth response 1, Cell migration, Proliferation, Paracrine actions, MAPK pathway, Hair growth, Regeneration

## Abstract

**Background:**

LL-37 is a naturally occurring antimicrobial peptide found in the wound bed and assists wound repair. No published study has characterized the role of LL-37 in the function(s) of human mesenchymal stem cells (MSCs). This study investigated the functions of adipose-derived stromal/stem cells (ASCs) activated by LL-37 by performing both in vitro assays with cultured cells and in vivo assays with C57BL/6 mice with hair loss.

**Methods:**

Human ASCs were isolated from healthy donors with written informed consent. To examine the effects of LL-37 on ASC function, cell proliferation and migration were measured by a cell counting kit (CCK-8) and a Transwell migration assay. Early growth response 1 (EGR1) mRNA expression was determined by microarray and real-time PCR analyses. The protein levels of EGR1 and regenerative factors were analyzed by specific enzyme-linked immunosorbent assays and western blotting.

**Results:**

LL-37 treatment enhanced the proliferation and migration of human ASCs expressing formyl peptide receptor like-1. Microarray and real-time PCR data showed that EGR1 expression was rapidly and significantly increased by LL-37 treatment. LL-37 treatment also enhanced the production of EGR1. Moreover, small interfering RNA-mediated knockdown of EGR1 inhibited LL-37-enhanced ASC proliferation and migration. Activation of mitogen-activated protein kinases (MAPKs) was essential not only for LL-37-enhanced ASC proliferation and migration but also EGR1 expression; treatment with a specific inhibitor of extracellular signal-regulated kinase, p38, or c-Jun N-terminal kinase blocked the stimulatory effect of LL-37. EGR1 has a strong paracrine capability and can influence angiogenic factors in ASCs; therefore, we evaluated the secretion levels of vascular endothelial growth factor, thymosin beta-4, monocyte chemoattractant protein-1, and stromal cell-derived factor-1. LL-37 treatment increased the secretion of these regenerative factors. Moreover, treatment with the conditioned medium of ASCs pre-activated with LL-37 strongly promoted hair growth in vivo.

**Conclusions:**

These findings show that LL-37 increases EGR1 expression and MAPK activation, and that preconditioning of ASCs with LL-37 has a strong potential to promote hair growth in vivo. This study correlates LL-37 with MSC functions (specifically those of ASCs), including cell expansion, cell migration, and paracrine actions, which may be useful in terms of implantation for tissue regeneration.

**Electronic supplementary material:**

The online version of this article (doi:10.1186/s13287-016-0313-4) contains supplementary material, which is available to authorized users.

## Background

Cell therapy using adult multipotent stromal cells or mesenchymal stem cells (MSCs) is clinically used to repair and regenerate various damaged tissues [1, 2]. Adipose-derived stromal/stem cells (ASCs) are multipotent mesenchymal cells isolated from adipose tissue and are accessible, abundant, and self-replenishing [3]. These cells are also capable of differentiating into multiple mesenchymal lineages, including osteoblasts, adipocytes, chondrocytes, and other types of cells. The differentiation, proliferation, and direct migration of MSCs into local damaged tissue undergoing regeneration are regarded as the primary mechanisms underlying the actions of MSCs [3, 4]. Besides these actions, the strong paracrine effects of various growth factors and cytokines secreted by MSCs is a key mechanism underlying MSC-mediated tissue regeneration and repair [5, 6]. Therefore, it is important to understand the molecular mechanism controlling the machinery that underlies these strong paracrine effects in MSCs.

Early growth response 1 (EGR1), a member of the immediate-early gene family, encodes a zinc finger transcription factor and is rapidly induced by mitogens and growth factors [7]. Once induced, EGR1 plays a pivotal role in the expression and production of various growth factors, cytokines, and other bioactive molecules, resulting in the stimulation of cellular functions for tissue repair and regeneration. Interestingly, EGR1 might be an activation marker of human MSCs, which highly express the *EGR1* gene, and have multiple roles involving angiogenesis and mitogenesis [8, 9]. Strong induction of EGR1 is mediated by the mitogen-activated protein kinase (MAPK) pathway, a crucial signaling pathway associated with cell migration and proliferation [7, 10].

LL-37 is a naturally occurring 37-amino acid sequence synthesized from the C-terminus of human cationic antimicrobial protein 18 (hCAP-18) [11] and is widely found in various body fluids and cell types including epithelial cells and immune cells [12, 13]. Secretion of LL-37/hCAP-18 is significantly elevated at the wound bed, where this peptide demonstrates proliferative, angiogenic, and immunomodulatory activities through the MAPK pathway [14]. Besides participating in innate host defense [11, 15], this peptide also has wound-healing effects [16, 17] and is a potent chemoattractant for various cell types including immune cells through activation of formyl peptide receptor like-1 (FPRL1), its main receptor [18]. A recent study by Krasnodembskaya et al. showed that human MSCs possess direct antimicrobial activity, which is mediated in part by secretion of the human cathelicidin hCAP-18/LL-37 [19].

Many studies reported ASC-mediated tissue regeneration in various damaged tissues [1, 20] and LL-37 is an important mediator of the repair and regeneration of wounds, bones, islets, and other damaged tissues [16, 21, 22]. However, the precise effect of LL-37 on adjacent human ASCs has not been identified. In the present study, we hypothesized that LL-37 enhances their therapeutic potential by activating ASCs via EGR1 and MAPK signaling. Our findings indicate that LL-37 may be used as a preconditioning agent before ASC transplantation for tissue regeneration.

## Methods

### Cell culture

Subcutaneous adipose tissue was obtained during elective surgeries with written informed consent, as approved by the Samsung Medical Center Institutional Review Board. All donors were < 40 years old and did not have diabetes or acute inflammation. The mean body mass index of the donors was 25.2 ± 3.64. Human ASCs were isolated according to a previous protocol [23] and cultured in low-glucose Dulbecco’s modified Eagle’s medium supplemented with 10 % fetal bovine serum, 100 U/mL penicillin, and 100 μg/mL streptomycin at 37 °C in a humidified atmosphere containing 5 % CO_2_. ASCs were characterized by the presence of the cell surface markers CD73, CD90, and CD105 and the absence of CD11b, CD34, CD45, and HLA-DR [24].

### Cell viability and proliferation assays

Cells were treated with human LL-37 (Phoenix Pharmaceuticals, USA) for 48 hr under serum deprivation conditions. Cell viability was determined by Trypan blue staining. Cell proliferation was measured with the cell counting kit (CCK)-8 according to the manufacturer’s protocol (Dojindo, Japan). ASCs (5 × 10^3^ cells/well) were treated with 2.5–20 μg/mL LL-37 for 24 and 48 hr prior to adding CCK-8 solution. Absorbance at 450 nm was determined with a multi-plate reader (Molecular Devices, CA, USA).

### Migration assay

A cell migration assay was performed using Transwell plates (8 μm pore size; Costar, Corning, NY, USA) according to a previous study [25]. Briefly, ASCs were suspended in serum-free medium and 100 μL of the cell suspension (7 × 10^5^ cells/mL) was added to each upper well. LL-37 at the indicated concentrations (5, 10, and 20 μg/mL) was placed in the lower wells of a 24-well tissue culture plate. After incubation for 6 hr at 37 °C, cells that had migrated were stained with 0.15 % crystal violet and counted in five random microscopy fields using the Scanscope scanning system (Aperio Scanscope, CA, USA).

### Small interfering RNA transfection

Cells were transfected with EGR1-targeting small interfering RNA (siRNA) (Santa Cruz Biotechnology, USA) or negative control siRNA (Bioneer, Daejeon, Korea) using Lipofectamine RNAi (Invitrogen, CA, USA). Briefly, when cells reached 60–70 % confluency, siRNA (final concentration, 100 nM) was combined with Lipofectamine RNAi and allowed to complex for 20 min. The transfection mixture was then applied to ASCs and incubated for 6 hr at 37 °C. Subsequently, cells were maintained in complete medium for 36 hr before being subjected to the cell migration assay and enzyme-linked immunosorbent assays (ELISAs).

### Western blot analysis

Total protein from cell lysates was separated by SDS-PAGE and transferred to a nitrocellulose membrane, which was incubated with the corresponding primary antibodies (anti-EGR1 (Santa Cruz Biotechnology) and anti-GAPDH (Cell Signaling, USA)) overnight at 4 °C. Washed membranes were incubated for 1 hr with a horseradish peroxidase-conjugated anti-mouse secondary antibody. Bands were visualized using an enhanced chemiluminescence detection system (Amersham Biosciences, Piscataway, NJ, USA). The band intensities were quantified using TotalLab software (UK).

### Fluorescence-activated cell sorting

FPRL1 expression in ASCs from four donors was evaluated by surface staining. Cells were washed with fluorescence-activated cell sorting (FACS) buffer, stained with a mouse anti-FPRL1 antibody (R&D Systems, USA), and then stained with anti-mouse IgG-FITC (Sigma, USA). Labeled cells were measured with a FACSCalibur instrument (Becton Dickinson Biosciences, CA, USA) and analyzed using the Win MDI program (Win MDI version 2.8).

### Immunostaining

Immunofluorescence analysis was performed as previously described [23]. Briefly, ASCs (5 × 10^3^ cells/well) were seeded on four-well Lab-Tek II chamber slides (Nalge Nunc International, IL, USA), treated with LL-37 for 48 hr, fixed, and permeabilized for 20 min with 0.1 % Triton X-100 prepared in phosphate-buffered saline. After washing, cells were blocked and incubated with an anti-proliferating cell nuclear antigen (PCNA) antibody (Abcam, Cambridgeshire, UK) for 1 hr. Next, cells were incubated with goat anti-mouse IgG-Alexa Fluor 488 (Invitrogen) for 30 min at 37 °C. A nucleic acid dye (DAPI; 0.5 μg/mL) was added to stain the nuclei. PCNA immunofluorescence was detected with an LSM700 confocal microscope system (Carl Zeiss, NY, USA; 400× objective).

### ELISA

Cells were plated in six-well plates (300,000 cells/well) and treated with 20 μg/mL LL-37 in serum-free medium. After 48 hr, the culture medium was collected and ELISAs were performed for thymosin beta-4 (TB4; Immune Diagnostics, Canada), vascular endothelial growth factor (VEGF; Invitrogen), monocyte chemoattractant protein-1 (MCP-1; eBioscience, USA), stromal cell-derived factor-1 (SDF-1; RayBiotech, USA), and EGR1 (EIAab, China), in accordance with the manufacturers’ recommendations. For detection of EGR1, the cells were lysed by the addition of 200 μL of cell lysis buffer after LL-37 treatment for 6 hr.

### Real-time PCR

Total RNA was extracted using TRIzol reagent (Invitrogen). After removing possible DNA contamination by DNAse treatment of the extracted RNA, cDNA was synthesized using 2 μg of total RNA and SuperScript II reverse transcriptase according to the manufacturer’s instructions. The primers used are provided in Additional file 1: Table S1. For real-time PCR, quantitative PCR was performed using the 7900 Real-Time PCR System (Applied Biosystems, Foster City, CA, USA) and the Power SYBR Green qPCR Master Mix Kit (Life Technologies, CA, USA). The cycling profile for real-time PCR (50 cycles) was as follows: 95 °C for 10 min, 95 °C for 15 sec, and 60 °C for 60 sec. The comparative threshold cycle (Ct) method (i.e., 2^-^ΔΔCt) was used to calculate fold amplification.

### Microarray

The microarray data are accessible in the Gene Expression Omnibus (GEO) database under accession number GSE76392. For the microarray, human ASCs were treated with 20 μg/mL LL-37 for 1 hr. Each total RNA sample (200 ng) was labeled and amplified using the Low Input Quick Amp Labeling Kit (Agilent Technologies, CA, USA). Cy3-labeled aRNAs were resuspended in 100 μL of hybridization solution (Agilent Technologies). Labeled aRNAs were placed on the Agilent SurePrint G3 Human GE 4x44K array (Agilent Technologies) and covered with a Gasket 8-plex slide (Agilent Technologies). Slides were hybridized for 17 hr at 65 °C. The hybridized slides were sequentially washed in 2× SSC containing 0.1 % SDS for 2 min, 1× SSC for 3 min, and 0.2× SSC for 2 min at room temperature. Finally, the slides were centrifuged at 3000 rpm for 20 sec to dry. Arrays were analyzed using an Agilent scanner with associated software. Gene expression levels were calculated with Feature Extraction v10.7.3.1 (Agilent Technologies). The relative signal intensities of each gene were generated using the Robust Multi-Array Average algorithm. Data were processed based on the quantile normalization method using GeneSpring GX 13.0 (Agilent Technologies). This normalization method aims to make the distribution of intensities for each array in a set of arrays the same. The normalized and log-transformed intensity values were analyzed using GeneSpring GX 13.0. Fold-change filters included the requirement that the genes be present in at least 200 % of controls for upregulated genes and less than 50 % of controls for downregulated genes. Hierarchical cluster analysis was conducted using the Cluster 3.0 program, the Euclidean distance, and average linkage algorithm.

### Animals and in vivo hair growth test

Five-week-old male C57BL/6 mice were purchased from SLC Inc. (Haruno, Japan) and allowed to adapt to their new environment for 2 weeks. The fur on the backs of 7-week-old mice (*n* = 14) was shaved with hair clippers and removed with hair removal cream. Conditioned medium (CM) was topically applied daily for up to 18 days. Pigment darkening and the hair growth rate were monitored every 3 days for 2 weeks. Hair growth was evaluated by three independent dermatological scientists. The hair growth score was determined using the method described by Vegesna et al. [26, 27]. The animal experiments were approved by the Institutional Animal Care and Use Committee of Samsung Medical Center (approval number: SMC-IACUC-2013-0103-003) and all experiments followed regulatory standards.

### Plasmid construction and selection of stably transfected cell lines

hEGR1 was subcloned into the *Hind* III/*Xho* I sites of pcDNA3.1(+). After digestion of the pcDNA3.1(+)-hEGR1 plasmid with the restriction enzymes, the resulting *hEGR1* gene along with the *BamH*I and *Sfi*I sites was inserted into the multiple cloning site of the expression plasmid pLenti6/V5-D-TOPO (Invitrogen), which contains a cytomegalovirus promoter upstream of the inserted gene. The resulting plasmid was named pLenti6/V5-hEGR1.

The lentiviral expression system based on four plasmids was obtained from Invitrogen. Briefly, 2.5 μg of pLP1, 2.5 μg of pLP2, 2.5 μg of pLP/VSVG, and 2.5 μg of the lentiviral vector pLenti6/V5-hCAMP were co-transfected with Fugene6 (Roche) into HEK293T cells cultured in 10 cm plates. The medium was changed every 24 hr. After 48 hr, supernatants were pooled, filtered through a 0.45-μm filter, and centrifuged at 6000 rpm at 4 °C for 16 hr. The pellet was resuspended in 1 mL of complete medium and stored at −80 °C. The resulting recombinant lentivirus was added to ASCs cultured in 10 cm plates. At 48 hr after seeding, the medium was replaced with complete medium containing 10 μg/mL blasticidin as a selective agent. At 18 days after transfection, selected cell colonies were split into 60 mm petri dishes. Isolated cell colonies were cultured and expanded.

### Statistical analysis

Statistical significance was estimated using the Student’s *t*-test. Mean differences were considered to be significant when *P* < 0.05.

## Results

### LL-37 increases the proliferation and migration of human ASCs in a dose-dependent manner

**Fig. 1:**
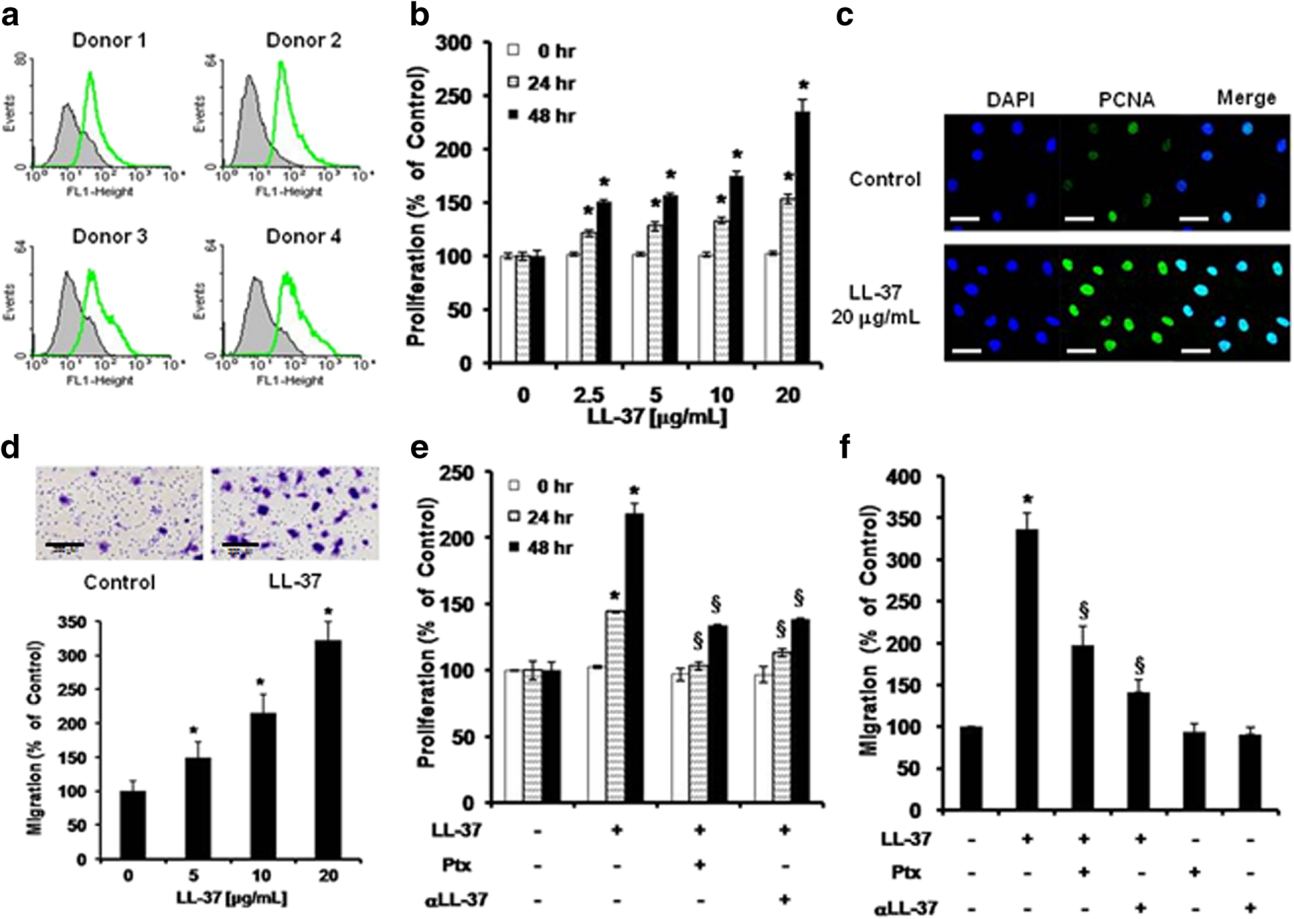
Effects of LL-37 on the proliferation and migration of human ASCs. **a** FPRL1 expression on ASCs from four donors, as analyzed by flow cytometry. **b** Proliferating cells were measured at 24 and 48 hr using the CCK-8 assay. A representative experiment of three independent experiments is shown. *Bars* represent the mean ± SD. *, *P* < 0.01 vs. control at 24 or 48 hr. **c** Immunostaining was performed and visualized using a confocal microscope. *Bar* = 100 μm. **d** ASCs were seeded in the upper wells and the lower wells were treated with 5, 10, and 20 μg/mL LL-37 for 6 hr. A migration assay was performed using Transwell chambers. Cells that migrated were counted using Scanscope (images show samples treated with 20 μg/mL LL-37). A representative experiment from three independent experiments is shown. *Bars* represent the mean ± SEM. *, *P* < 0.05 vs. control. **e** ASC proliferation was analyzed after pretreatment of cells with Ptx or an anti-LL-37 neutralizing antibody (αLL-37) with or without LL-37. **f** ASC migration was analyzed after pretreatment of cells with Ptx or αLL-37 prior to LL-37 treatment. Values represent the mean ± SD of three independent experiments. *, *P* < 0.01 vs. control; ^§^, *P* < 0.01 vs. LL-37-treated cells. *ASCs* adipose-derived stromal/stem cells, *FPRL1* formyl peptide receptor like-1, *Ptx* pertussis toxin

LL-37 reportedly activates functions such as proliferation and migration through FPRL1, a G-protein-coupled receptor, in many cell types [18]; therefore, several donor pools of ASCs were examined for expression of FPRL1. Flow cytometric data confirmed that FPRL1 was expressed on ASCs in each donor pool (Fig. 1a).

To investigate the role of LL-37 in ASC proliferation, we examined the effect of LL-37 on the proliferative ability of ASCs using the CCK-8 assay. LL-37 treatment markedly stimulated ASC proliferation in a dose-dependent manner without causing cytotoxicity (Fig. 1b and Additional file 2: Figure S1a). Furthermore, we determined the number of proliferating cells by performing immunofluorescence staining of PCNA, a nuclear protein associated with cell proliferation. The proportion of ASCs with positively stained nuclei (green fluorescence) was markedly enhanced by LL-37 treatment (Fig. 1c).

To investigate the effects of LL-37 on ASC migration, a Transwell migration assay was performed. LL-37 treatment rapidly increased human ASC migration within 6 hr; this effect was dose-dependent, with maximal stimulation at 20 μg/mL among the concentrations tested (Fig. 1d). ASCs were pretreated with pertussis toxin (Ptx), a Gαi inhibitor, or a neutralizing anti-LL-37 antibody (αLL-37) before activation with LL-37. Ptx or αLL-37 treatment prior to LL-37 stimulation significantly inhibited ASC migration and proliferation (Fig. 1e and f). Taken together, these data suggest that LL-37 induces ASC proliferation and migration through the Gαi-coupled receptor FPRL1.

### EGR1 is critical for LL-37-enhanced ASC migration and proliferation

**Fig. 2:**
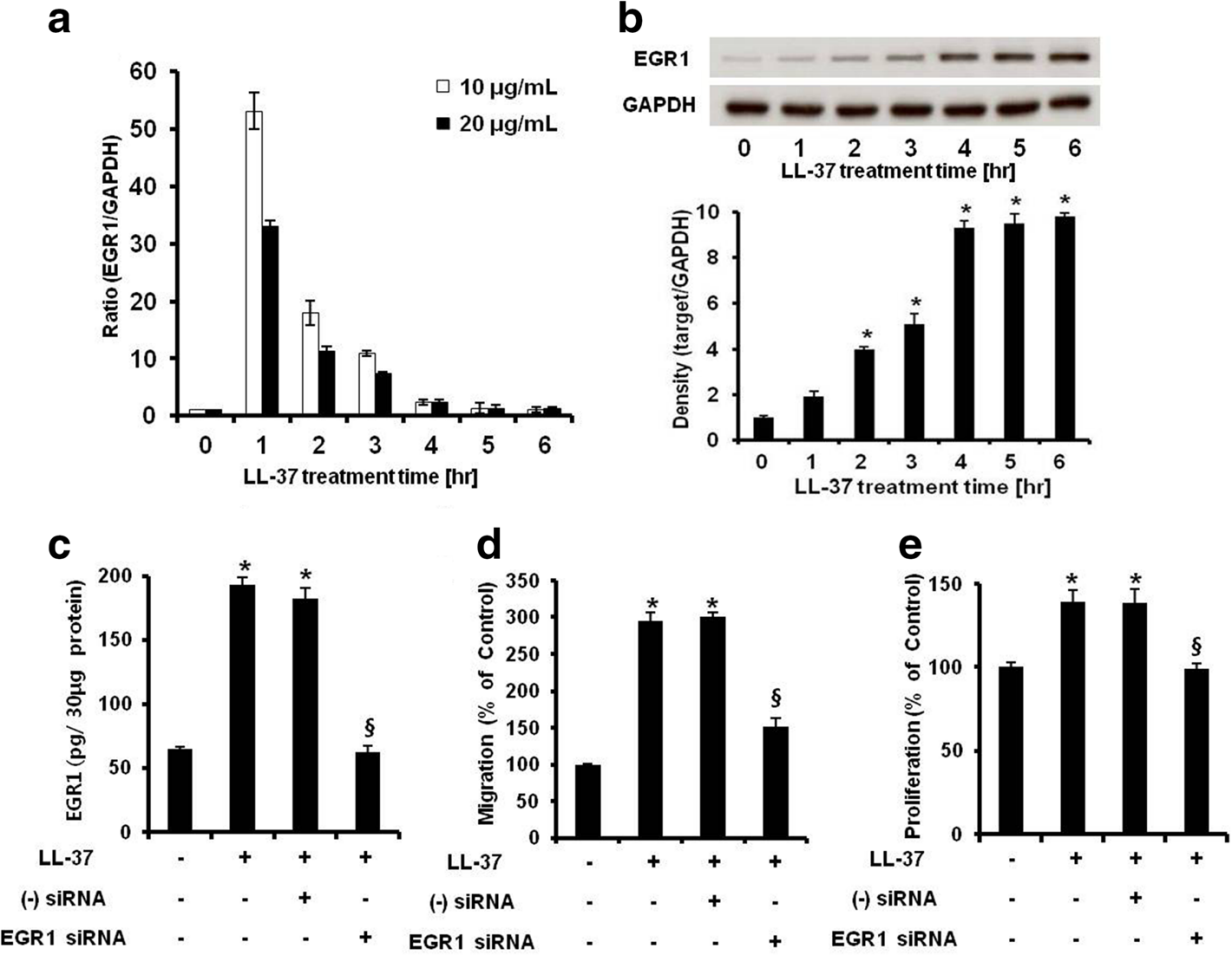
EGR1 is critical for human LL-37-enhanced ASC migration and proliferation. **a** EGR1 mRNA expression was determined by real-time PCR. EGR1 mRNA was detected after treatment with 10 and 20 μg/mL LL-37 for 0–6 hr. **b** ASCs were incubated with 20 μg/mL LL-37 for 0–6 hr. Cell lysates were collected for western blot analysis. The EGR1 protein level was increased by LL-37 treatment in a time-dependent manner. **c** ASCs were treated with LL-37 and transfected with siRNA targeting EGR1 or a negative control sequence. After stabilization, cells were collected and lysed by the addition of 200 μL of cell lysis buffer. EGR1-targeting siRNA transfection was quantified by performing an EGR1-specific ELISA of cell lysates. **d** Cells that migrated after EGR1-targeting siRNA transfection were imaged by microscopy and counted using Scanscope. Results are expressed as the mean ± SD of three independent experiments. *, *P* < 0.01 vs. control; ^§^, *P* < 0.01 vs. LL-37-treated cells transfected with negative control siRNA. **e** The effects on human ASC proliferation were similar to those on migration. A representative experiment from three independent experiments is shown. *Bars* represent the mean ± SD. *, *P* < 0.01 vs. control; ^§^, *P* < 0.01 vs. LL-37-treated cells transfected with negative control siRNA. *EGR1* early growth response 1, *ASC* adipose-derived stromal/stem cells, *siRNA* small interfering RNA, *ELISA* enzyme-linked immunosorbent assay

We investigated the target genes of LL-37 that induce ASC proliferation and migration using the Agilent human 4x44K array, a human signaling pathway finder. Microarray analysis showed that LL-37 treatment significantly increased EGR1 expression by 18.47-fold (Table 1 and Additional file 3: Table S2). LL-37 treatment also increased the levels of several genes (including *EGR2*, early growth response 2; *KLF10*, Krupple-like factor 10; *FOS*; and *CTGF*, connective tissue growth factor) linked to various functions, such as the cell cycle, cell migration, cell proliferation, and transcription. To confirm the effect of LL-37 treatment on EGR1 expression in ASCs, cells were treated with 10 or 20 μg/mL LL-37 for different amounts of time and then real-time PCR analysis was performed. LL-37 treatment significantly increased the EGR1 mRNA level, which peaked at 1 hr and returned to the basal level at about 6 hr (Fig. 2a). Because LL-37 treatment considerably increased the mRNA level of EGR1, we attempted to ascertain whether it also increased the protein level of EGR1 via western blot analysis. LL-37 treatment rapidly increased the protein level of EGR1 in ASCs in a time-dependent manner (Fig. 2b). In addition to western blot analysis, ELISA analysis of cell lysates demonstrated that EGR1 production was significantly increased by LL-37 treatment (Additional file 2: Figure S1b)

**Table 1:** List of genes up-regulated by LL-37

Gene name	Accession No	Fold increase	Classification
Early growth response 1 (*EGR1*)	NM_001964	18.47	Transcription, Immune response
Early growth response 2 (*EGR2*)	NM_000399	6.13	Transcription
High mobility group AT-hook 2 (*HMGA2*)	NM_003483	4.72	Transcription
Response gene to complement 32 (*RGC32*)	NM_014059	4.33	Cell cycle
Solute carrier family 20 (phosphate transporter) (*SLC20A1*)	NM_005415	4.19	Signal transduction
Dual specificity phosphatase 1 (*DUSP1*)	NM_004417	3.94	Cell cycle
Dual specificity phosphatase 6 (*DUSP6*)	NM_001946	3.74	Cell cycle, Signal transduction
Connective tissue growth factor (*CTGF*)	NM_001901	3.71	Cell migration, Cell growth, Cell adhesion, Signal transduction
Kruppel-like factor 10 (*KLF10*)	NM_005655	3.31	Transcription, Signal transduction, Cell proliferation
Dachsous 1 (Drosophila) (*DCHS1*)	NM_003737	3.05	Cell adhesion
Tumor necrosis factor receptor superfamily, member 12A (*TNFRSF12A*)	NM_016639	2.98	Apoptosis, Cell adhesion
Pleckstrin homology-like domain, family A, member 2 (*PHLDA2*)	NM_003311	2.94	Apoptosis
v-fos FBJ murine osteosarcoma viral oncogene homolog (*FOS*)	NM_005252	2.80	Transcription, Inflammatory response, Immune response
Tribbles homolog 2 (Drosophila) (*TRIB2*)	NM_021643	2.56	Cell adhesion
Tubulin, beta 2C (*TUBB2C*)	NM_006088	2.52	Transport, Apoptosis
Ras association (RalGDS/AF-6) and pleckstrin homology domains 1 (*RAPH1*)	NM_213589	2.46	Signal transduction
Myeloid cell leukemia sequence 1 (BCL2-related) (*MCL1*)	NM_021960	2.34	Apoptosis
SH2B adaptor protein 3 (*SH2B3*)	NM_005475	2.26	Cell cycle, Signal transduction
Cysteine-rich, angiogenic inducer, 61 (*CYR61*)	NM_001554	2.19	Cell adhesion, Cell proliferation
Sphingosine kinase 1 (*SPHK1*)	NM_021972	2.10	Cell migration, Apoptosis, Cell cycle, Cell proliferation
Ras association (RalGDS/AF-6) and pleckstrin homology domains 1 (*RAPH1*)	NM_213589	2.01	Signal transduction

The fold increase indicates the increase in expression in comparison to control cells, as determined by a human cDNA microarray

Next, to study the involvement of EGR1 in LL-37-enhanced ASC proliferation and migration, ASCs were transfected with EGR1-targeting siRNA. We confirmed *EGR1* gene silencing at the protein level (Fig. 2c). LL-37-enhanced proliferation and migration of ASCs were markedly attenuated by EGR1-targeting siRNA transfection (Fig. 2d and e). These data suggest that EGR1 is important for LL-37-enhanced migration and proliferation of ASCs.

### Involvement of the MAPK pathway in LL-37-induced ASC proliferation and migration

**Fig. 3:**
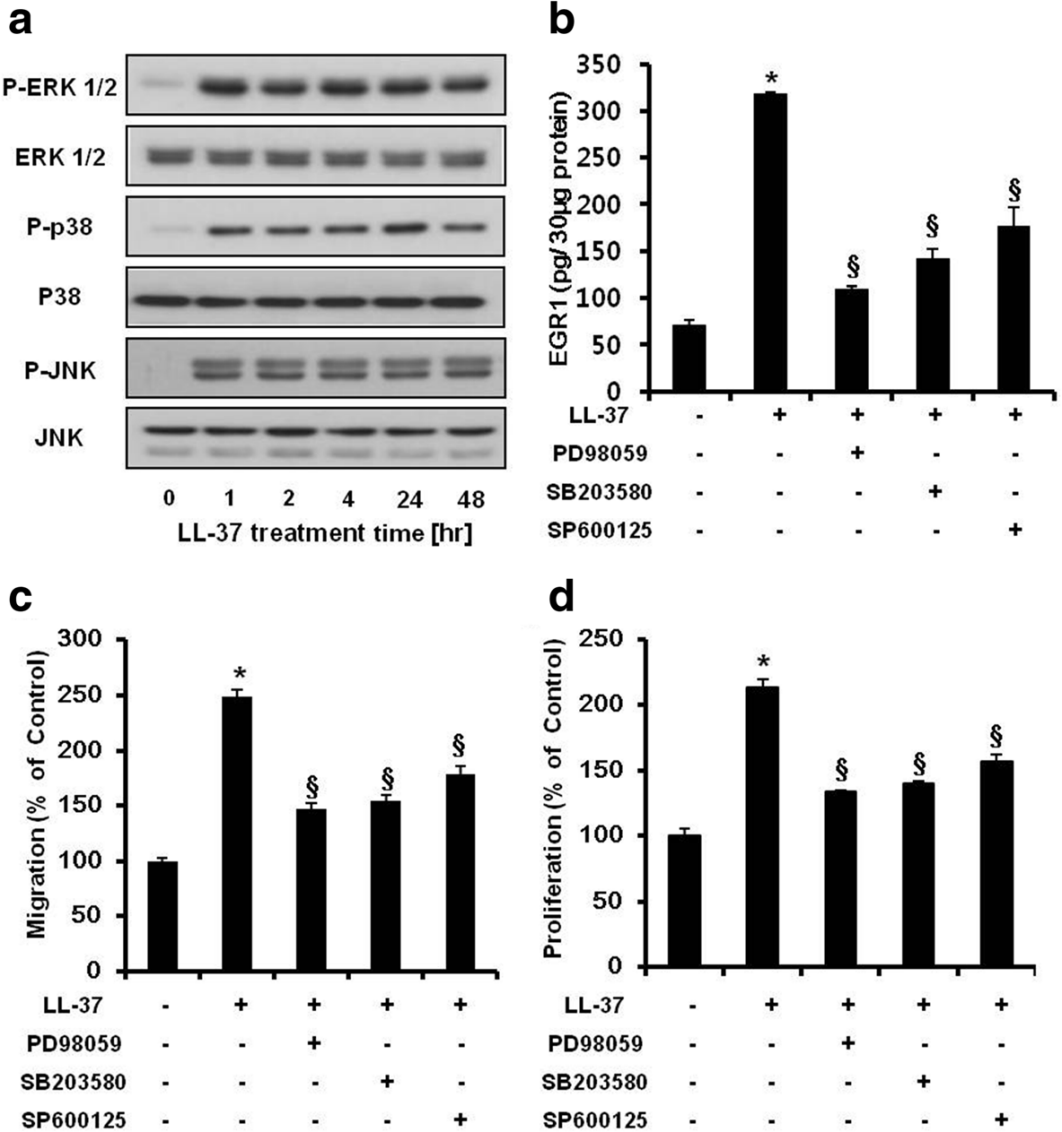
Involvement of the MAPK pathway in LL-37-induced ASC proliferation and migration. **a** Human ASCs were treated with 20 μg/mL LL-37 for 1, 2, 4, 24, and 48 hr. After cell lysis, the levels of phosphorylated ERK1/2, p38, and JNK were determined by western blot analysis. The levels of total ERK1/2, p38, and JNK were used to confirm equal loading of the cell lysates. LL-37 treatment significantly increased the phosphorylation of ERK1/2, p38, and JNK. **b** ASCs were pretreated with or without a specific inhibitor of ERK1/2 (PD98059), p38 (SB203580), or JNK (SP600125) for 1 hr and then treated with 20 μg/mL LL-37. EGR1 protein levels in cell lysates were analyzed by an EGR1-specific ELISA. **c** LL-37-enhanced migration decreased in human ASCs treated with PD98059, SB203580, or SP600125. **d** LL-37-enhanced ASC proliferation was inhibited by treatment with specific inhibitors of ERK1/2, p38, or JNK (similar to ASC migration). A representative experiment from three independent experiments is shown. *Bars* represent the mean ± SD. *, *P* < 0.01 vs. control; ^§^, *P* < 0.01 vs. LL-37-treated ASCs. *MAPK* mitogen-activated protein kinase, *ASCs* adipose-derived stromal/stem cells, *EGR1* early growth response-1

The MAPK pathway plays an important role in the migration and proliferation of MSCs and cancer cells [10, 28]. To elucidate the signaling mechanism involved in LL-37-induced ASC proliferation and migration in detail, we examined the effect of LL-37 on the MAPK pathway in human ASCs. Treatment with LL-37 significantly increased the levels of phosphorylated extracellular signal-regulated kinase (ERK) 1/2, p38, and c-Jun N-terminal kinase (JNK), but did not change the total levels of these proteins (Fig. 3a). To further determine the involvement of kinase phosphorylation in the increased migration, proliferation, and EGR1 production of ASCs, cells were treated with LL-37 in the presence and absence of a specific inhibitor of ERK (PD98059), p38 (SB203580), or JNK (SP600125). All these MAPK inhibitors (PD98059, SB203580, and SP600125) significantly reduced LL-37-induced EGR1 production (Fig. 3b). In addition, each of the inhibitors attenuated the LL-37-induced increase in ASC migration and proliferation (Fig. 3c and d). Taken together, these data suggest that: 1) LL-37 induces multiple signaling pathways, including those linked with ERK, p38, and JNK phosphorylation; and 2) these kinases are involved in the regulation of human ASC migration and proliferation. These results suggest that LL-37 stimulates ASC function through a MAPK-dependent mechanism.

### LL-37 treatment enhances growth factor production in human ASCs, and treatment with the CM of ASCs pre-activated with LL-37 stimulates hair regeneration in vivo

**Fig. 4:**
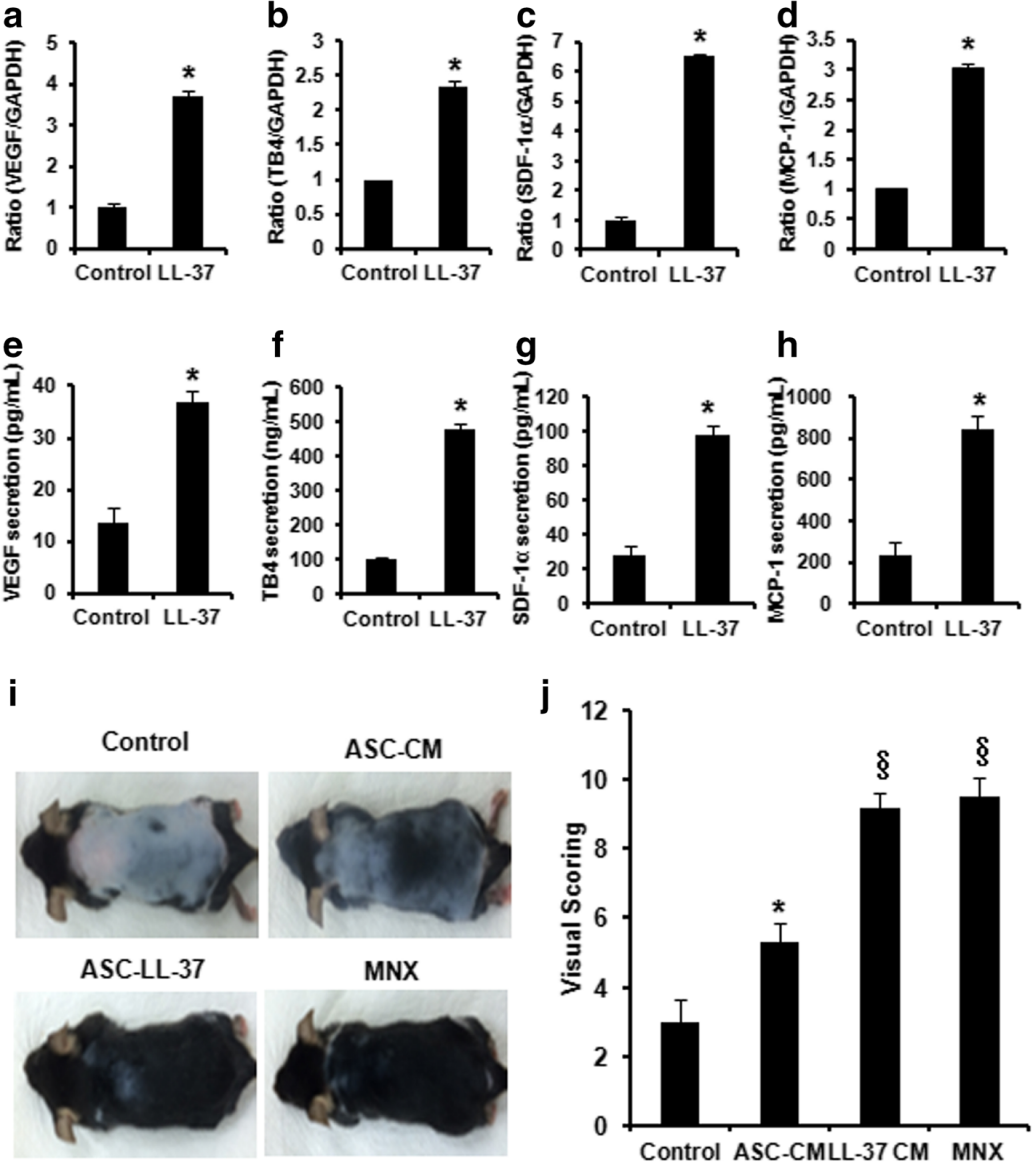
Preconditioning with LL-37 stimulates hair regeneration in vivo and production of regenerative factors in vitro. **a** VEGF, (**b**) TB4, (**c**) SDF-1α, and (**d**) MCP-1 mRNA expression was determined by real-time PCR. **e** VEGF, (**f**) TB4, (**g**) SDF-1α, and (**h**) MCP-1 proteins were detected using the supernatants of confluent cell cultures. ASCs were treated with 20 μg/mL LL-37 for 48 hr, and then the protein levels of VEGF, TB4, SDF-1α, and MCP-1 were analyzed using specific ELISAs. A representative experiment from three independent experiments is shown. *Bars* represent the mean ± SD. *, *P* < 0.05 vs. control. **i** Hair was removed from the backs of C57BL/6 mice and the hair growth rate was monitored for 3 weeks. CM of human ASCs pretreated with or without LL-37 (20 μg/mL) was topically applied daily for up to 18 days to mice with hair loss. Gross views observed by photographs. **j** Hair growth was scored as described in the Materials and methods section. *, *P* < 0.05 for control vs. group treated with ASC CM. ^§^, *P* < 0.05 for group treated with ASC CM vs. group treated with CM of ASCs pre-activated with LL-37. *VEGF* vascular endothelial growth factor, *TB4* thymosin beta-4, *SDF*-*1*α stromal cell-derived factor-1α, *MCP*-*1* monocyte chemoattractant protein-1, *ASC* adipose-derived stromal/stem cell, *ELISA* enzyme-linked immunosorbent assay, *CM* conditioned medium

It was recently reported that EGR1 has a strong paracrine capability and can influence angiogenic factors in ASCs [8, 29]; therefore, we first examined whether LL-37 regulates the expression of various regenerative growth factors and bioactive molecules in ASCs. LL-37 treatment considerably upregulated the mRNA expression of VEGF, TB4, MCP-1, and SDF-1 (Fig. 4a–d). We next confirmed the direct secretion of these growth factors by ASCs using ELISAs. The secretion levels of VEGF, TB4, MCP-1, and SDF-1 were significantly increased by LL-37 treatment (Fig. 4e–h).

To further evaluate the paracrine effects of LL-37 in vivo, the hair growth of C57BL/6 mice with hair loss was tested in vivo. We compared hair growth after topically applying CM of ASCs pre-activated with or without LL-37 (20 μg/mL). After 2 weeks, the hair growth score was higher in the group treated with CM of ASCs than in the negative control group treated with medium alone. Moreover, the hair growth score was higher in the group treated with CM of ASCs pre-activated with LL-37 than in the group treated with CM of non-activated ASCs (Fig. 4i and j), with dark pigmentation and hair regeneration observed on the initially pink hairless skin. Treatment with minoxidil (MNX), which is widely used to treat hair loss, was also effective for hair regeneration. Specifically, 95–100 % of hair was regenerated to full length in the group treated with CM of ASCs pre-activated with LL-37 in comparison to the MNX-treated group, whereas the level of regeneration was lower in the group treated with CM of non-activated ASCs (30–35 %; Fig. 4i). Additionally, to elucidate the biological function of EGR1 in vitro and in vivo, EGR1 was lentivirally introduced into human ASCs. Cell lines stably expressing the control vector and EGR1 were designated pLenti6/V5-D-TOPO and pLenti6/V5-hEGR1, respectively. As expected, the secretion levels of VEGF, TB4, MCP-1, and SDF-1 were significantly higher in the medium of cells overexpressing EGR1 than in the medium of wild type (WT) cells or those expressing the control vector (Additional file 2: Figure S2a–2d). To confirm the paracrine effects of stable EGR1-overexpressing cells in vivo, we performed an additional investigation of hair regeneration. Mice were treated with CM of WT ASCs, ASCs expressing the control vector, or EGR1-overexpressing ASCs. Compared with CM of WT ASCs and ASCs expressing the control vector, treatment with CM of EGR1-overexpressing ASCs considerably increased hair regeneration (Additional file 2: Figure S2e and 2f).

Taken together, LL-37 might mediate paracrine actions by stimulating the secretion of growth factors such as VEGF, TB4, SDF-1, and MCP-1 by ASCs. In addition, CM from ASCs pre-activated with LL-37 and EGR1-overexpressing cells strongly promotes hair growth in vivo.

## Discussion

Many recent studies report clinical trials of ASCs (or CM from ASCs) for the treatment of vascular injury and for the promotion of bone, skin, and hair regeneration [1, 2, 30]. However, the regenerative effects of MSCs are limited because they vary according to the donor, the donor’s age, the sampling site, and the culture techniques and conditions. Therefore, it is essential to develop an activator of MSCs. LL-37 is a naturally occurring antimicrobial peptide found in the wound bed, where it assists wound repair and regeneration [15–17]; it is important to elucidate the relationship between LL-37 and adjacent ASCs for tissue regeneration and repair. Therefore, we investigated the functions of ASCs activated by LL-37 exposure.

We demonstrated that human ASCs expressed FPRL1 and that LL-37 treatment significantly enhanced the proliferation and migration of these cells. Next, we searched for the factor(s) that underlies LL-37-enhanced migration and proliferation of ASCs. Initially, we thought that TB4 and MCP-1 were candidates for such factors because LL-37 treatment considerably enhanced their production (Fig. 4f and h), which facilitated ASC migration (unpublished data). However, these are not the main factors, although this is only one possible mechanism underlying LL-37-enhanced migration of ASCs. This is because LL-37 treatment rapidly promoted the migration of human ASCs within 6 hr (Fig. 1d), whereas TB4 and MCP-1 production was noticeably changed much later, after about 24 hr (Fig. 4f and h). Our microarray and real-time PCR data showed that EGR1 mRNA expression rapidly responded to LL-37 stimulation within 1 hr (Fig. 2a and Table 1). Moreover, western blot and ELISA analyses revealed that LL-37 treatment also significantly increased EGR1 protein levels by about 5–10-fold at 6 hr (Fig. 2b and Additional file 2: Figure S1b), which regulated ASC migration (Fig. 2d). Some previous studies reported that EGR1 mRNA was rapidly induced within 1–2 hr and that EGR1 protein was also relatively rapidly induced at the same time [29, 31]. Interestingly, our data showed that EGR1 protein induction was a little sluggish, although EGR1 mRNA expression was immediately increased within 1 hr. However, as in other reports, EGR1 protein was slowly induced at 5–6 hr [32–34]. It is possible that the optimal time of EGR1 induction differs according to the type of stimulus (e.g., cytokine, growth factor, and oxidative stress) or the cell type. A recent study by Min et al. suggested that EGR1 controls hematopoietic stem cell migration and expansion [35]. Similarly, our data showed that EGR1-targeting siRNA transfection inhibited LL-37-enhanced proliferation and migration of ASCs (Fig. 2c–e). Thus, these results suggest that EGR1 is important for the regulation of LL-37-enhanced proliferation and migration of human ASCs.

In this study, LL-37 promoted the expansion and migration of human ASCs at all concentrations, but was most effective at 20 μg/mL (Fig. 1b and d). Interestingly, EGR1 expression was highest in ASCs treated with a lower concentration (10 μg/mL) of LL-37 (Fig. 2a), suggesting that, in addition to EGR1, other factors and mechanisms mediate the effects of LL-37 on the proliferation and migration of human ASCs. For example, LL-37 treatment also considerably increased interleukin (IL)-8 expression and secretion in human ASCs (Additional file 2: Figure S3a and S3b). We previously demonstrated that IL-8 induced by TB4 plays a key role in ASC proliferation [23]. Moreover, IL-8 stimulated ASC migration, and IL-8 knockdown using IL-8-specific siRNA inhibited cell migration (unpublished data). IL-8 promotes angiogenesis and migration of bone marrow-derived MSCs [36], and may also affect multiple functions of human ASCs, including their proliferation and migration. Further investigations are necessary to clarify the other mechanisms underlying LL-37-induced proliferation and migration of human ASCs and the factors involved. Taken together, these data suggest that EGR1 is an important, but not the only, factor for LL-37-mediated ASC migration and proliferation.

It was recently reported that EGR1 has a strong paracrine capability and can influence angiogenic factors in ASCs [8, 29]; therefore, we investigated the enhanced secretion of TB4, VEGF, MCP-1, and SDF-1 in response to LL-37 stimulation (Fig. 4a–h). These growth factors and cytokines are associated with the regeneration and repair of various tissues, including skin, bone, and skeletal muscle [37–40]. Among them, TB4 directly stimulates hair growth and proliferation of hair follicle dermal papilla cells. Furthermore, TB4 accelerates hair growth via activation, migration, and differentiation of hair follicle stem cells [38, 41]. Recently, VEGF was shown to stimulate hair growth by facilitating the supply of nutrients to the hair follicle, increasing the follicular diameter [42, 43]. Based on these reports, TB4 and VEGF are regarded as the main factors in the hair growth promotion effect of the CM of LL-37-activated ASCs. Interestingly, one of these regenerative factors, MCP-1, is a pro-inflammatory cytokine. However, it also promotes healing in diabetic wounds, dental pulp, and skeletal muscle by increasing cell migration, angiogenesis, or macrophage infiltration, leading to a higher regenerative potential [39, 44, 45]. Therefore, MCP-1 seems to be a key cytokine mediating regenerative effects as well as a pro-inflammatory cytokine, and its action might depend on the microenvironment in the human body. LL-37-enhanced regenerative factors also influence their microenvironment through paracrine actions [46]. In support of this, CM from ASCs pre-activated with LL-37 strongly promoted hair growth as efficiently as MNX (95–100 % of hair), which is a commercially available treatment for hair loss (Fig. 4i and j). Besides these paracrine actions, we previously demonstrated that the SDF-1/CXCR4 axis is essential for human dermal fibroblast (HDF) migration, resulting in wound-healing effects [25]. This suggests that CXCR4-expressing HDFs can migrate along the concentration gradient of SDF-1 secreted by human ASCs in response to LL-37 at wound sites, leading to wound repair and regeneration. Liu et al. and Neuhaus et al. reported that SDF-1 also enhances EGR1 expression in endothelial cells and angiogenesis in ischemic regions [47, 48]. Based on these reports, LL-37-induced SDF-1 might also enhance EGR1 expression in human ASCs. Therefore, it is reasonable to conclude that LL-37 can gradually accelerate other mechanisms via autocrine loops and paracrine actions on adjacent cells such as HDFs. Taken together, LL-37 may activate human ASCs via autocrine and paracrine actions.

## Conclusions

**Fig. 5:**
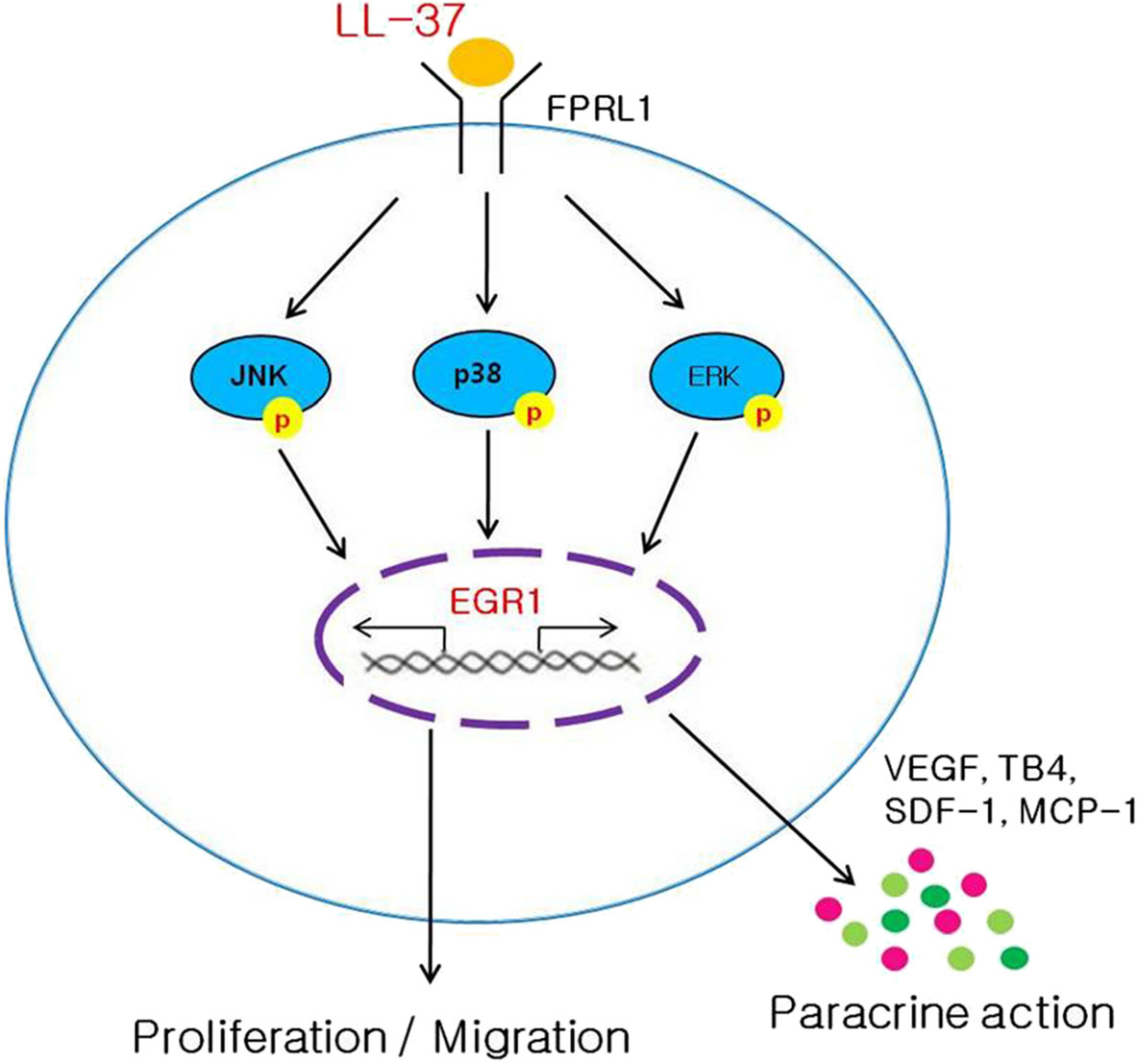
Scheme representing the functions of ASCs after LL-37 treatment. LL-37 enhances ASC proliferation and migration via the EGR1 and MAPK pathways. Furthermore, LL-37 stimulates the secretion of growth factors such as VEGF, TB4, SDF-1, and MCP-1 in human ASCs. CM from ASCs preconditioned with LL-37 strongly promotes hair growth in vivo. LL-37 can be modulated to activate human ASCs and may provide a therapeutic approach for tissue regeneration by enforcing the functions of ASCs (e.g., expansion, migration, and paracrine actions). *ASCs* adipose-derived stromal/stem cells, *EGR1* early growth response 1, *MAPK* mitogen-activated protein kinase, *VEGF* vascular endothelial growth factor, *TB4* thymosin beta-4, *SDF*-*1*α stromal cell-derived factor-1α, *MCP*-*1* monocyte chemoattractant protein-1, *CM* conditioned medium

In summary, these results demonstrate that LL-37 enhances ASC proliferation and migration via the EGR1 and MAPK pathways (Fig. 5). Furthermore, LL-37 stimulates the secretion of growth factors such as VEGF, TB4, SDF-1, and MCP-1. CM from ASCs preconditioned with LL-37 strongly promotes hair growth in vivo. Therefore, LL-37 can be modulated to activate human ASCs and may provide a therapeutic approach to tissue regeneration by enforcing the functions of ASCs (e.g., expansion, migration, and paracrine actions).

## Additional files

Additional file 1: Table S1. List of primers used for real-time PCR. (PDF 106 kb) Additional file 2: Figure S1. Effects of LL-37 on the viability and EGR1 production of human ASCs. **Figure S2**. EGR1-overexpressing ASCs secrete higher levels of the regenerative factors VEGF, TB4, SDF-1α, and MCP-1, and the CM of these cells stimulates hair regeneration in vivo. **Figure S3**. LL-37 increases the expression and production of IL-8 in human ASCs. (PDF 267 kb) Additional file 3: Table S2. List of upregulated genes in ASCs exposed to LL-37 by microarray analysis. (XLS 45 kb)

## Abbreviations

ASC: adipose-derived stromal/stem cell
CCK-8: cell counting kit-8
CM: conditioned medium
CTGF: connective tissue growth factor
EGR1: early growth response 1
EGR2: early growth response 2
ELISA: enzyme-linked immunosorbent assay
ERK: extracellular signal-regulated kinase
FACS: fluorescence-activated cell sorting
FPRL1: formyl peptide receptor like-1
hCAP-18: human cationic antimicrobial protein 18
HDF: human dermal fibroblast
IL: interleukin
JNK: c-Jun N-terminal kinase
KLF10: Krupple-like factor 10
MAPK: mitogen-activated protein kinase
MCP-1: monocyte chemoattractant protein-1
MNX: minoxidil
MSC: mesenchymal stem cell
PCNA: proliferating cell nuclear antigen
Ptx: pertussis toxin
SDF-1: stromal cell-derived factor-1
siRNA: small interfering RNA
TB4: thymosin beta-4
VEGF: vascular endothelial growth factor
WT: wild type
αLL-37: anti-LL-37 antibody
