# Supplementary material for: LL-37 stimulates the functions of adipose-derived stromal/stem cells via early growth response 1 and the MAPK pathway
Source: Stem Cell Res Ther. 2016 Apr 19;7:58. doi: 10.1186/s13287-016-0313-4 (PMC4837546; doi:10.1186/s13287-016-0313-4)
Supplement: Additional file 1: Table S1. — List of primers used for real-time PCR. (PDF 106 kb) [file 13287_2016_313_MOESM1_ESM.pdf]

Table S1. List of primers used for real-time PCR

Target gene	Forward	Reverse
EGR1	5’-CTT CAA CCC TCA GGC GGA CA-3’	5’- GGA AAA GCG GCC AGT ATA GGT-3’
VEGF	5’-CCC ACT GAG TCC AAC AT-3’	5’- TTT CTT GCG CTT TCG TTT TT-3’
TB4	5’- TGC TTG CTT CTC CTG TTC AA-3’	5’- ACA AAC CCG ATA TGG CTG AGA TCG AG-3’
SDF-1α	5’-CTA CTC TCT CCC CGA CTC CG-3’	5’-AAG CAG GGG GAC CAT TAC AC-3’
MCP-1	5’-CCC CAG TCA CCT GCT GTT AT-3’;	5’-TGG AAT CCT GAA CCC ACT TC-3’
GAPDH	5’-ATC ACC ATC TTC CAG GAG CGA-3’	5’-TTC TCC ATG GTG GTG AAG ACG-3’
