# Supplementary material for: LL-37 stimulates the functions of adipose-derived stromal/stem cells via early growth response 1 and the MAPK pathway
Source: Stem Cell Res Ther. 2016 Apr 19;7:58. doi: 10.1186/s13287-016-0313-4 (PMC4837546; doi:10.1186/s13287-016-0313-4)
Supplement: Additional file 2: Figure S1. — Effects of LL-37 on the viability and EGR1 production of human ASCs. Figure S2. EGR1-overexpressing ASCs secrete higher levels of the regenerative factors VEGF, TB4, SDF-1α, and MCP-1, and the CM of these cells stimulates hair regeneration in vivo. Figure S3. LL-37 increases the expression and production of IL-8 in human ASCs. (PDF 267 kb) [file 13287_2016_313_MOESM2_ESM.pdf]

Fig. S1

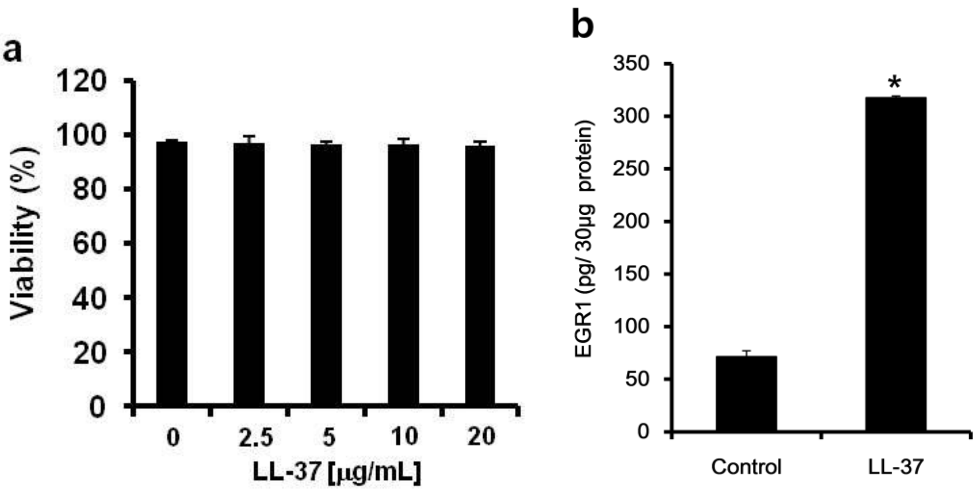

Fig. S1: Effects of LL-37 on the viability and EGR1 production of human ASCs. (a) The viability of human ASCs treated with 2.5, 5, 10, and 20 µg/mL LL-37 was evaluated. (b) ASCs were treated with 20 µg/mL LL-37 for 6 hr and EGR1 protein levels in cell lysates were quantified by an EGR1-specific ELISA. A representative experiment from three independent experiments is shown. Bars represent the mean ± SD. *, $P < 0.01$ *vs*. control.

Fig. S2

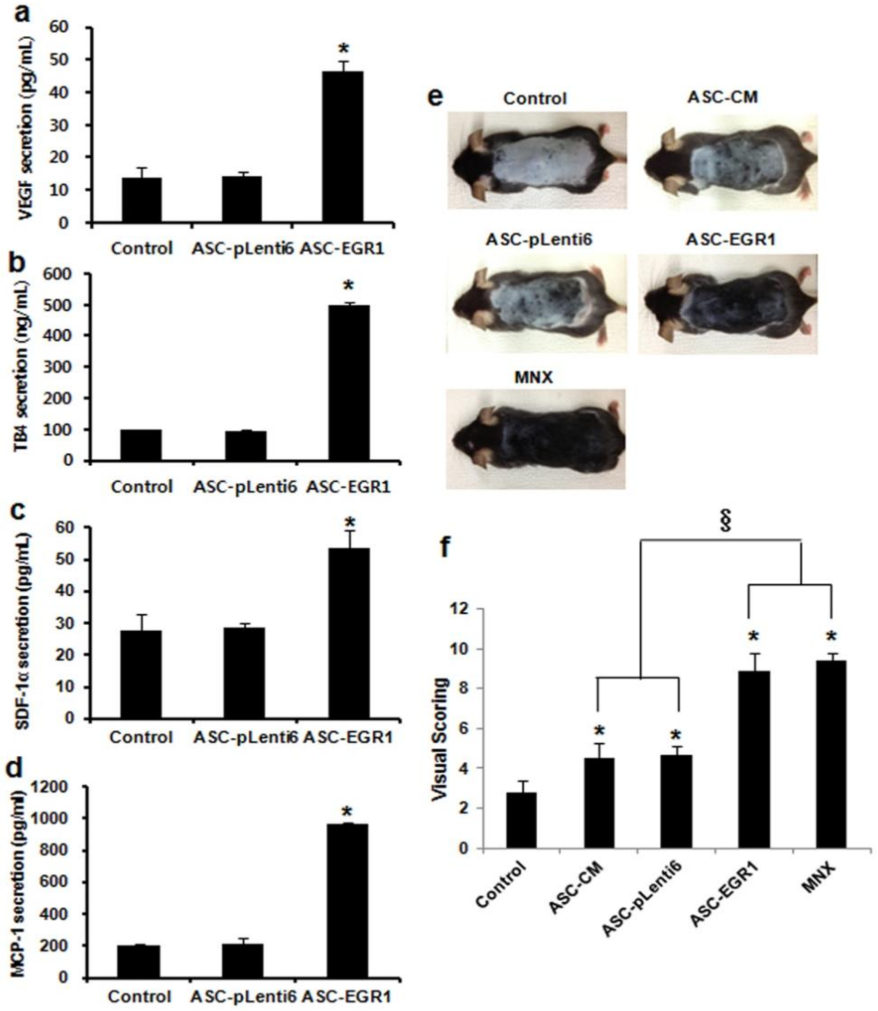

Fig. S2: EGR1-overexpressing ASCs secrete higher levels of the regenerative factors VEGF, TB4, SDF-1α, and MCP-1, and the CM of these cells stimulates hair regeneration *in vivo*.

Stable cell lines were generated using the control vector pLenti6/V5-D-TOPO and the EGR1-harboring vector pLenti6/V5-hEGR1. (a) VEGF, (b) TB4, (c) SDF-1α, and (d) MCP-1 proteins were detected in the CM of confluent cell cultures using specific ELISAs. Bars represent the mean ± SD of three independent experiments. *, $P < 0.05$ *vs.* control. CM of WT ASCs, ASCs expressing the control vector (ASC-pLenti6), or EGR1-overexpressing ASCs (ASC-EGR1) was topically applied daily for

up to 18 days to mice with hair loss. (e) Gross views observed by photographs. (f) Hair growth was scored as described in the Materials and methods section. *, $P < 0.05$ for negative control group *vs.* group treated with CM of WT ASCs or ASC-pLenti6. [§], $P < 0.05$ for group treated with CM of ASC-pLenti6 *vs.* group treated with CM of ASC-EGR1.

Fig. S3

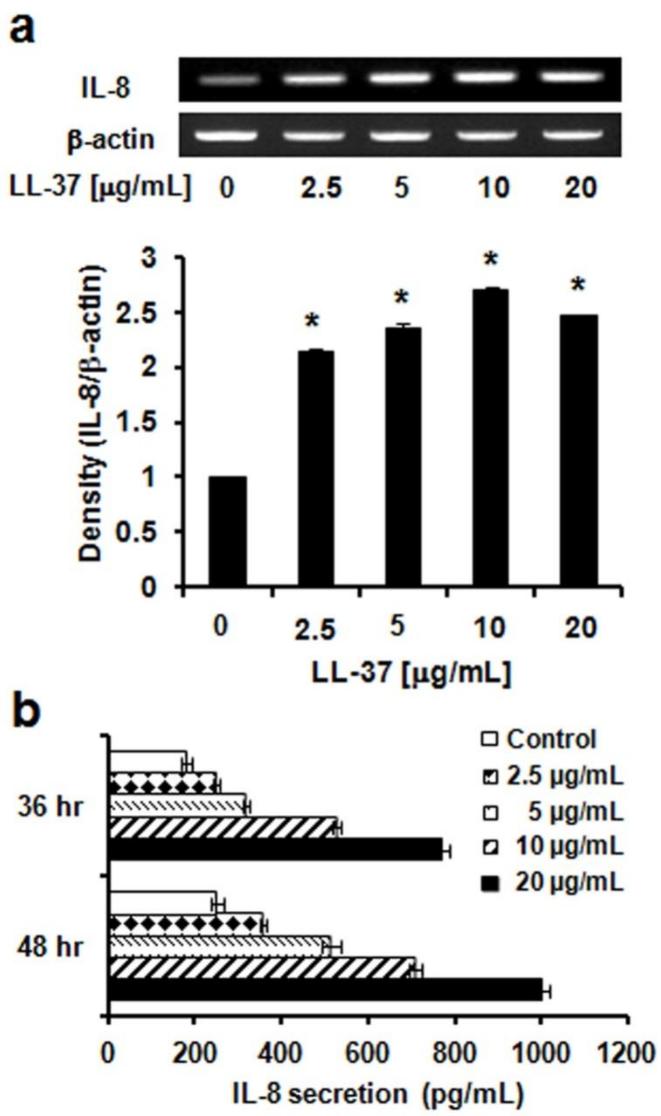

Fig. S3: LL-37 increases the expression and production of IL-8 in human ASCs.

(a) IL-8 mRNA expression was determined by RT-PCR. IL-8 mRNA was detected after treatment with 2.5, 5, 10, and 20 μg/mL LL-37 for 24 hr. Bars represent the mean ± SD. *, $P < 0.05$ *vs.* control. (b) IL-8 proteins were detected in the CM of confluent cell cultures. ASCs were incubated with 2.5, 5, 10, and 20 μg/mL LL-37 for 36 and 48 hr, and then the protein levels of IL-8 were analyzed using specific ELISAs. Bars represent the mean ± SD of three independent experiments.
